# Molecular detection and quantification of the *Striga* seedbank in agricultural soils

**DOI:** 10.1111/wre.12535

**Published:** 2022-04-29

**Authors:** Getahun Mitiku, Dominika Rybka, Paulien Klein‐Gunnewiek, Taye Tessema, Jos M. Raaijmakers, Desalegn W. Etalo

**Affiliations:** ^1^ Department of Microbial Ecology Netherlands Institute of Ecology, NIOO‐KNAW Wageningen The Netherlands; ^2^ Ethiopian Institute of Agricultural Research Addis Ababa Ethiopia; ^3^ Institute of Biology Leiden University Leiden The Netherlands; ^4^ Laboratory of Phytopathology Wageningen University and Research Wageningen The Netherlands

**Keywords:** mapping, qPCR, sorghum field, *Striga* seed density, sub‐Saharan Africa, weed seed

## Abstract

*Striga hermonthica* (Del.) Benth is a devastating parasitic weed in Sub‐Saharan Africa (SSA) and its soil seedbank is the major factor contributing to its prevalence and persistence. To date, there is a little information on the *Striga* seedbank density in agricultural fields in SSA due to the lack of reliable detection and quantification methods. We developed a high‐throughput method that combines density‐ and size‐based separation techniques with quantitative polymerase chain reaction (qPCR)‐based detection of *Striga* seeds in soil. The method was optimised and validated by introducing increasing numbers of *Striga* seeds in two physicochemically different *Striga*‐free agricultural soils. The results showed that as little as one seed of *S. hermonthica* per 150 g of soil could be detected. This technique was subsequently tested on soil samples of 48 sorghum fields from different agro‐ecological zones in Ethiopia to map the geospatial distribution of the *Striga* seedbank along a trajectory of more than 1500 km. Considerable variation in *Striga* seed densities was observed. *Striga* seeds were detectable in 75% of the field soils with densities up to 86 seeds per 150 g of soil. The *Striga* seed density in soil and the number of emerged *Striga* plants in the field showed a non‐linear relationship. In conclusion, the method developed allows for accurate mapping of the *Striga* seedbank in physicochemically diverse SSA field soils and can be used to assess the impact of management strategies on *Striga* seedbank dynamics.

## 1 INTRODUCTION

*Striga* is one of the major genera of parasitic plants with considerable yield‐limiting effects on diverse staple crops such as sorghum, maize, pearl millet and upland rice in semi‐arid and sub‐humid zones of Africa. Outside Africa, the occurrence of *Striga* is also reported in Asia, USA, Arabian Peninsula and Australia (Ejeta, 2007; Eplee, 1992; Musselman & Ayensu, 1984; Nail et al., 2014). Though *Striga* is less problematic in developed countries, it is still a severe problem leading to food insecurity for resource poor smallholder farmers in the majority of African and Asian countries. More than 50 species of *Striga* have been reported across the globe with *S. hermonthica, S. asiatica*, *S. gesneroides, S. aspera* and *S. forbesii* as the most common and destructive in cultivated cereal and legume crops (Parker, 2009; Scholes & Press, 2008). It is estimated that two‐thirds of the total area of cereals and legumes in SSA are infested with *Striga* and its spread has accelerated at an alarming rate (Parker, 2012). The annual yield losses due to *Striga* were estimated at US $7 billion in SSA, posing a major threat to the livelihood of over 300 million people (Ejeta, 2007). Ethiopia is one of the epicentres of *Striga* infestation in SSA and crop losses of 65%–100% are commonly reported for different sorghum growing regions of the country (Abate et al., 2014; Bayu et al., 2005; Tesso et al., 2007).

Management of *Striga* in many parts of the world is constrained largely by *Striga* seeds residing for multiple years in the soil, also referred to as the ‘seedbank’. For instance, *Striga* produces 3000–84 000 tiny seeds (0.2–0.3 mm, 4–7 μg per seed) per plant (Delft et al., 1997; Mourik, 2007; Webb & Smith, 1996), which survive in soil for at least 2 years (Bebawi et al., 1984). However, other reports suggested significant decline in seed viability in natural soil due to potential microbial activity (Delft et al., 1997; Gbèhounou et al., 2003; Mourik et al., 2003). Managing *Striga* requires a better understanding of seedbank replenishment and depletion, also referred to as the seedbank dynamics. Replenishment encompasses seed production by mature *Striga* plants and ‘immigration’ of seeds from neighbouring field soils via wind and movements of human, animal, runoff water, crop residue and agricultural implements. Seedbank depletion is caused by suicidal germination (i.e. germination in absence of a host plant), pathogen infection, seed predation, seed aging, and ‘emigration’ of seeds to neighbouring fields (Mourik, 2007). Hence, a methodology that allows accurate detection and quantification of the actual *Striga* seed density in agricultural fields is of paramount importance in the management of *Striga* in general and for understanding the mechanisms underlying seedbank dynamics in particular.

So far, some attempts have been made to quantify the *Striga* seedbank in agricultural fields in different African countries (Abunyewa & Padi, 2003; Delft et al., 1997; Franke et al., 2006; Mourik, 2007; Oswald & Ransom, 2001; Sauerborn et al., 2003; Schulz et al., 2003). These studies employed manual seed–soil separation methods accompanied by counting of the *Striga* seeds under a microscope. By using such methods, up to 882 000 *S. hermonthica* seeds per square meter was recorded in Kenya following two cropping seasons in a field that was initially infested with 60 000 *Striga* seeds per square meter and in which, hosts were grown twice per year (Oswald & Ransom, 2001). Such an approach is time consuming and is often prone to biases and error considering the small size of the *Striga* seeds and the large diversity of physicochemical properties of the field soils. Due to the lack of fast and robust methods for detection and quantification of *Striga* seedbank, the assessment of emerged *Striga* plants in an area is often used as a proxy for seedbank density (Delft et al., 1997). However, the number of emerged *Striga* plant do not always reflect the true *Striga* seedbank density as substantial number of *Striga* seeds could remain dormant even under favourable conditions for germination. Hence, reliable detection and quantification of the *Striga* seedbank are crucial to understand the dynamics of this parasitic weed and to evaluate the effectiveness of management strategies (Mourik et al., 2008; Westerman et al., 2007).

The advancement of techniques to extract environmental DNA and RNA (eDNA and eRNA) from soils with different physicochemical characteristics followed by qPCR or sequencing has opened new means for sensitive and accurate detection and quantification of specific (micro) organisms. Such techniques have been used for several years to detect and quantify pathogenic microorganisms in order to deploy or optimise early measures to prevent disease outbreaks in farms (Ophel‐Keller et al., 2008; Taparia et al., 2020). Furthermore, integration of high‐throughput eDNA extraction and qPCR can maximise the number of samples that can be processed in a single day, reducing labour costs and turnaround times (Prider et al., 2013). Recently, the use of qPCR has received attention for determining the seedbank of weeds present in a soil. Examples include, the use of molecular markers and DNA‐based assays for the detection and identification of seeds of different species of the parasitic weeds *Orobanche* and *Phelipanche* (Aly et al., 2012; Dongo et al., 2012; Kirilova et al., 2019; Prider et al., 2013; Román et al., 2007). To date, however, there are no reports on high‐throughput molecular detection and quantification of *Striga* seeds in agricultural soils. We developed a high‐throughput molecular method for detection and quantification of the *Striga* seedbank in field soils. The method encompasses a density‐ and size‐based separation of *Striga* seeds from the soil matrix followed by eDNA extraction and qPCR‐based detection and quantification. The optimised protocol was then used to quantify and map the geospatial distribution of the *Striga* seedbank in sorghum field soils collected from different agro‐ecological zones in Ethiopia covering a trajectory of more than 1500 km and to relate the seed densities to number emerged *Striga* plants in these fields.

## 2 MATERIALS AND METHODS

### 2.1 Soil sampling and study areas

The soil samples were collected from naturally *Striga* infested sorghum fields in Amhara (North Shewa, South and North Wollo Zones) and Tigray (West, Central and South zones) and Oromia special zone (Kemise) of Ethiopia in October 2017 (Figure 4A). For representative soil sampling, sorghum fields with four categories (zero, low, medium and high) of *Striga* field infestation were randomly selected. These categories were determined based on the number of emerged *Striga* plants counted for four quadrants of 1 m × 1 m. Soil samples from the top layer (0–20 cm) around the root zone of the sorghum plant in these quadrants were sampled separately and later combined together to form one composite sample per field. Tools used for the sampling were washed with water and rinsed with 70% ethanol between successive samplings to avoid cross contamination of samples. In total, soil was sampled from 48 *Striga* infested sorghum fields covering a trajectory of more than 1500 km across different agro‐ecological zones in Ethiopia. For each field, soil was collected from four randomly selected spots. Among the 48 fields, four soil samples from push–pull demonstration sorghum fields in North Shewa, Kemise and West Hararghae Zones of Ethiopia were included to investigate the effect of push–pull technologies on the *Striga* seedbank density in agricultural fields. Soil samples were brought to the laboratory at Holeta National Agricultural Biotechnology Research Centre, air‐dried and sieved through a 4 mm mesh sieve to remove stones and plant debris and soils coming from different spots of the same field were mixed to constitute a composite sample representing each field. Furthermore, seven *Striga*‐free soil samples were collected from different parts of the Netherlands and used to investigate *Striga* DNA recovery and qPCR efficiency.

### 2.2 Selection of marker genes, primer design and specificity

From the Parasitic Plant Genome Project, PPGP website (http://ppgp.huck.psu.edu/), the *Striga hermonthica* StHe0GB1 transcriptome assembly was downloaded. We selected five genes (StHe0GB1_1, StHe0GB1_9, StHe0GB1_20, StHe0GB1_76 and StHe0GB1_93) with *Striga*‐specific sequences as a putative marker gene for *Striga* seed detection and quantification (Table S1). For these five genes, 14 primer pairs were designed targeting the *Striga*‐specific sequences. Using the NCBI primer blast Web tool and nr‐database, the specificity of the forward and reverse primers was validated in silico (data not shown). To validate the efficacy of these primers experimentally, we extracted DNA from ~6000 *Striga* seeds, from 100 mg of *Striga* ‐free Dutch agricultural soil spiked with ~6000 *Striga* seeds and 100 mg of the soil sample without *Striga* seeds (control). Samples were ground manually with mortar and pestle in liquid nitrogen and kept at −80°C until further use. The genomic DNA was extracted using DNeasy PowerSoil Kit (QIAGEN) according to the manufacturer's instructions. The DNA quality and quantity were determined using a NanoDrop spectrophotometer.

PCR was performed in 25 μl reaction volume using GoTaq hot start polymerase master mix (12.5 μl), primer mix (1 μl), template DNA (0.5 μl) and water (11 μl) on a thermocycler equipped with a heated lid. An initial denaturation for 2 min at 95°C, 35 cycles with 30 s at 95°C, 30 s at 50°C, 30 s at 72°C and a final elongation for 5 min at 72°C. The primer sets were also evaluated in qPCR. The 20 μl qPCR mixes constitute 4 μl of the template DNA, 10 μl of SYBR Green, 1 μl of each forward and reverse primer (10 ppm), 2 μl of BSA (4 mg/ml) and 2 μl Sigma water. Two annealing temperatures (56°C, 60°C) were tested to assess the sensitivity and specificity of the primers. Bio‐Rad qPCR machine was used with the following conditions: 3 min at 95°C followed by 35 amplification cycles of 5 s at 95°C, 15 s at 56°C or 60°C and 25 s for the final elongation at 72°C.

### 2.3 *Striga* seed separation from soil matrix

To reduce the influence of soil physicochemical properties on DNA recovery and qPCR efficiency, methods that separate *Striga* seeds from the bulk soil to enhance the detection and accurate quantification of *Striga* seeds in soil were investigated. As a first step, using two *Striga‐*free Dutch agricultural soil samples (D08 and D17) that have contrasting physicochemical properties, we optimised density‐based extraction by K_2_CO_3_ solution followed by size‐dependent separation by sieving method. Briefly, the samples were divided into three 250 ml centrifuge bottles with 50 g of soil sample suspended in 150 ml of 5.5 M K_2_CO_3_ solution. Then, the soil samples were dispersed by shaking at 250 rpm for 15 min followed by sonication for 15 min by using Bransonic® Ultrasonic Cleaner sonicator containing a RF frequency of 47 KHZ ± 6%. The dispersed soil samples were centrifuged at 5000 *g* for 5 min at room temperature by using high‐speed centrifuge. The *Striga* seeds and other lighter organic matter floated on the top of the supernatant, whereas the majority of the soil particles settled at the bottom. The supernatants from the three bottles of the same sample were collected into 1000 ml bottles and the process was repeated a second time to ensure full recovery of all the *Striga* seeds. Then, size‐dependent separation of the *Striga* seeds, smaller soil particles and organic debris from larger particles was performed by using two meshes (pore sizes 425 and 75 μm) arranged in successive order. The *Striga* seeds and smaller particles retained on 75 μm were dried at 35°C for 48 h and subjected to grinding and DNA extraction.

The efficiency of the density and size‐dependent method described above was assessed in proof‐of‐principle experiments involving introduction of known numbers of *S. hermonthica* seeds (0, 1, 3, 9, 27, 81 and 243 seeds) into 150 g of two soil samples (D08 and D17) with contrasting soil physicochemical properties. Then, the *Striga* seeds were re‐separated from 150 g of soil samples and were ground manually by mortar and pestle under liquid nitrogen. The genomic DNA was also extracted using DNAeasy PowerSoil Kit (QIAGEN) according to the manufacturer's instructions as indicated above. Then, qPCR was performed to assess the effectiveness of the recovery of the *Striga* seeds from the soil matrix.

### 2.4 Establishment of standard curves

Two standard curves based on different concentration of recombinant plasmid DNA (rpDNA) containing the marker gene (StHe0GB1_93) and genomic DNA samples from six densities of *Striga* seeds introduced in 150 g of *Striga*‐free agricultural soil were used to establish relationship between cycle of quantification (*Cq*) value and seed number when analysing the naturally infested Ethiopian soil samples.

#### 2.4.1 Recombinant plasmid DNA‐based standard curve

The marker gene was first cloned in pGEM®‐T Easy vector. Then, the vector containing the marker gene was transformed into the *E. coli* and positive colonies were identified using colony PCR and cultured in LB medium. The rpDNA was isolated and purified and the concentration of the rpDNA was determined. A five‐point ten‐fold serial dilutions (0.5 pg/μl to 0.00005 pg/μl) of the purified rpDNA were subjected for qPCR assay to establish the relationship between *Cq* values and the calculated gene copies of the marker gene.

An initial number of gene copies μl^−1^ of a single strand (ss)‐rpDNA (NGC ss‐rpDNA) was calculated from the initial DNA concentration of the rpDNA (0.5 pg/μl), the length of the plasmid containing the target gene (3535 bp), the number of targets per DNA fragment (*n*_target_ [2 copies]), the Avogadro constant (6.022 * 10^23^ bp mol^−1^) and the average weight of a double‐stranded base pair (660 g mol^−1^ = 6.6 * 10^11^ ng mol^−1^) (Equation 1) (Brankatschk et al., 2012).

(1) $$\mathrm{NGC}\;\mathrm{ss}-\text{rpDNA}=2*\frac{6.02\;x\;1023\;\left(\frac{\text{copy}}{\mathrm{mol}}\right)*\mathrm{DNA}\;\text{amount}\;\left(g\right)}{\mathrm{DNA}\;\text{length}\;\left(\mathrm{bp}\right)*660\;\left(\frac{\frac{g}{\mathrm{mol}}}{\mathrm{dp}}\right)}$$

The linear regression of the *Cq* value of each dilution versus their corresponding log_10_ gene copy (*N*_0 *Sample*_) was used to calculate the slope (b) and intercept (a) of the standard curve (Equation 2) (Brankatschk et al., 2012). The amplification efficiency (*E*) was calculated from the slope of the standard curve using Equation 3.

(2) $$\mathrm{Cq}\;\text{sample}=a+b*\log\;\left(\mathrm{No}\;\text{sample}\right)$$

(3) $$E=10^{\left(-\frac{1}{b}\right)}\;$$

#### 2.4.2 *Striga* seedbank density‐based standard curve

Another standard curve was also established from the genomic DNA extracted above from soil sample (D08) mixed with six densities of *Striga* seeds (1, 3, 9, 27, 81 and 243 seeds). The gene copies of each density of the seeds were calculated from the mean *Cq* value by using the regression formula generated above from rpDNA gene copies and the corresponding mean *Cq* value. Then, the relationship between the number of *Striga* seeds and the estimated gene copies was generated. Hence, this regression equation is used to convert the detected DNA of *Striga* seeds by qPCR to a quantified number of seeds in naturally infested soils.

The above Equation 2 was also rearranged and taken the reverse of *Log* of both sides to calculate the number of gene copies *Striga* seed DNA (NGC ssDNA) extracted from different densities of seed introduced in *Striga* free Dutch soil D08 as indicated in (Gallup, 2011).

(4) $$\mathrm{NGC}\;\text{ssDNA}={\left(10\right)}^{\left(\frac{\mathrm{Cq}\;\text{ssDNA}-a\;\text{rpDNA}}{b\;\text{rpDNA}}\right)}={\left(\text{EAMP rpDNA}\right)}^{\left(a\;\text{rpDNA}-\mathrm{Cq}\;\text{ssDNA}\right)}$$

The number of gene copies of *Striga* seed DNA extracted from naturally infested field soils (NGC ss DNA soil) was calculated per Equation 5 as described in (Gallup, 2011).

(5) $$\mathrm{NGC}\;\mathrm{ss}\;\mathrm{DNA}\;\text{soil}={\left(\text{EAMPssDNA}\right)}^{\left(a\;\text{rpDNA}\log_{(\text{EAMP rpDNA}}\text{EAMP ssDNA}\right)-\mathrm{Cq}\;\mathrm{ss}\;\mathrm{DNA}\;\text{soil})}$$

The standard curve that established a relationship between *Striga* seed number and gene copy created above from artificially contaminated soil samples with different densities of *Striga* seeds was used to extrapolate the number of *Striga* seeds in naturally infested soil samples from the mean *Cq* value‐gene copy relationship.

## 3 RESULTS

### 3.1 Selection of marker genes, primer design and specificity

**FIGURE 1:**
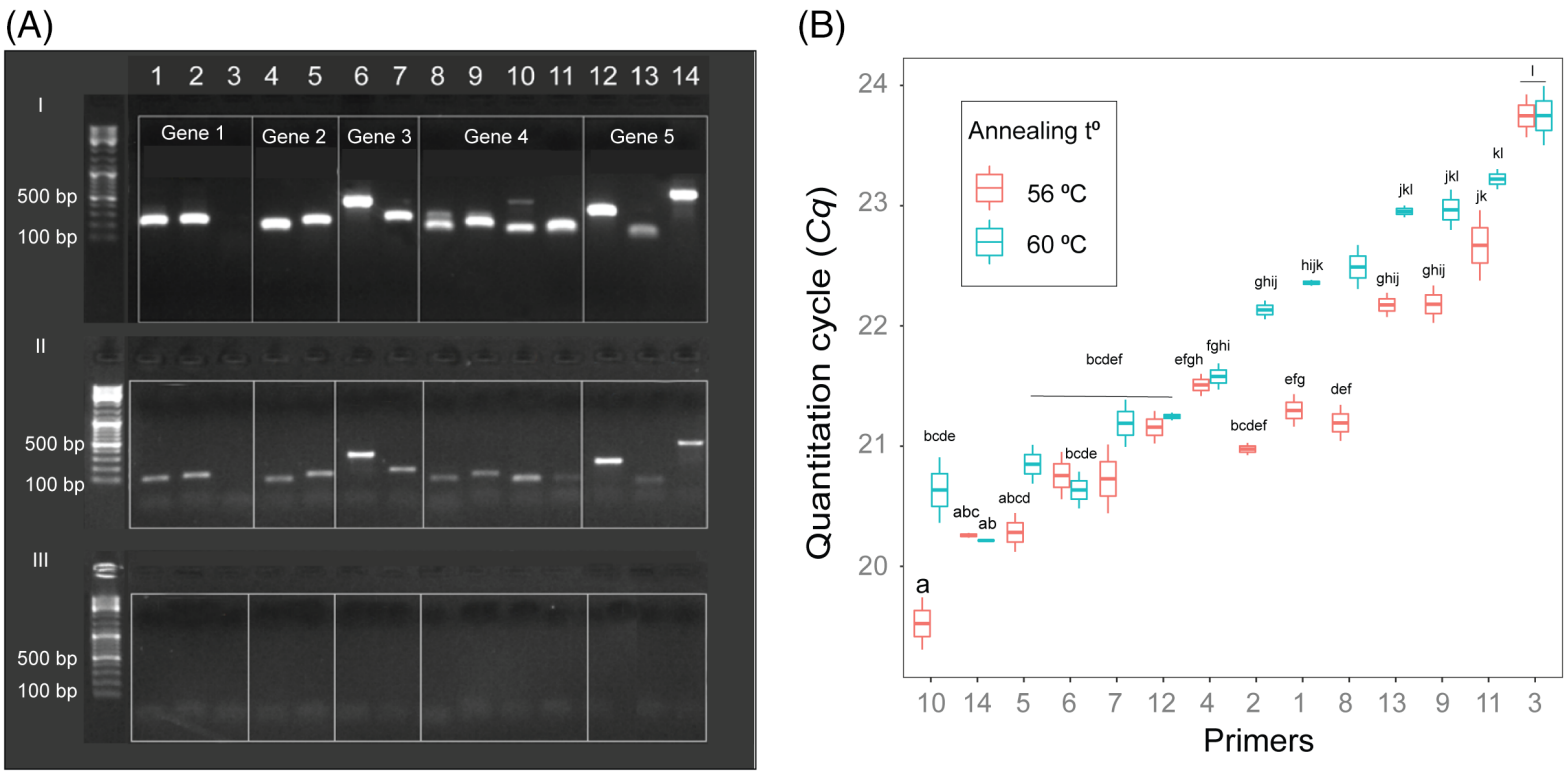
Molecular detection of *Striga* seeds in soil. (A) Gel electrophoresis of the PCR amplification products of five *Striga hermonthica* marker genes. A total of 14 primer sets were tested with 3 sets for gene 1 (StHe0GB1_1), 2 sets for gene 2 (StHe0GB1_9), 2 sets for gene 3 (StHe0GB1_2), 4 sets for gene 4 (StHe0GB1_76) and 3 sets for gene 5 (StHe0GB1_93)). The genomic DNA was extracted from (I) 50 mg *Striga* seeds, (II) 50 mg *Striga* seeds mixed in 100 mg of Dutch agricultural soil, and (III) 100 mg of Dutch agricultural soil (no *Striga* seed added; control). For the 14 primer sets, the sizes of the predicted PCR products are 145, 161, 170, 115, 157, 111, 200, 112, 154, 101, 100, 276, 70 and 520 base pairs (see Table S1). (B) Mean *Cq* values of the qPCR analysis with the 14 primer sets using DNA extracted from Dutch agricultural soil mixed with *S. hermonthica* seeds. The qPCR analysis was tested at two different annealing temperatures (56°C, 60°C). Mean *Cq* values (± SE) of three biological replicates (with two technical replicates per biological replicate) are shown. Different letters above each of the bars represent statistically significant differences (*p* < 0.05) between the *Cq* values of each of the 14 primer sets

To validate the efficacy of the primers experimentally, we extracted DNA from ~6000 *Striga* seeds, from *Striga*‐free Dutch agricultural soil spiked with ~6000 *Striga* seeds and the soil sample without *Striga* seeds (control). All primer sets, except set 3 (targeting StHe0GB1_1), resulted in the PCR product of the expected size (Table S1) for the samples containing the *Striga* seeds, whereas no amplification product was observed for any of the primer sets with DNA extracted from the soil samples without *Striga* seeds (Figure 1A).

Next, we tested the primer pairs in qPCR at two annealing temperatures (56 and 60°C) to determine sensitivity, specificity and stability of the primers. All primers amplified the genomic DNA of *Striga* seeds at both temperatures but with different sensitivity, specificity and stability (Figure 1B **)**. Primer set 14 (P14) targeting the StHe0GB1_93 gene showed high sensitivity as manifested by a low *Cq* value and single melting curve for both annealing temperatures (Figure 1B and Figure S2). Furthermore, P14 resulted in a PCR product of the expected size for five independent *S. hermonthica* ecotypes and one *S. asiatica* ecotype collected from different agroecological zones in Ethiopia (Figure S1). Hence, primer set P14 was selected for testing the specificity and sensitivity of PCR‐based detection and quantification of *Striga* seeds in soil samples.

### 3.2 Optimising DNA extraction and qPCR efficiency in different agricultural soils

**FIGURE 2:**
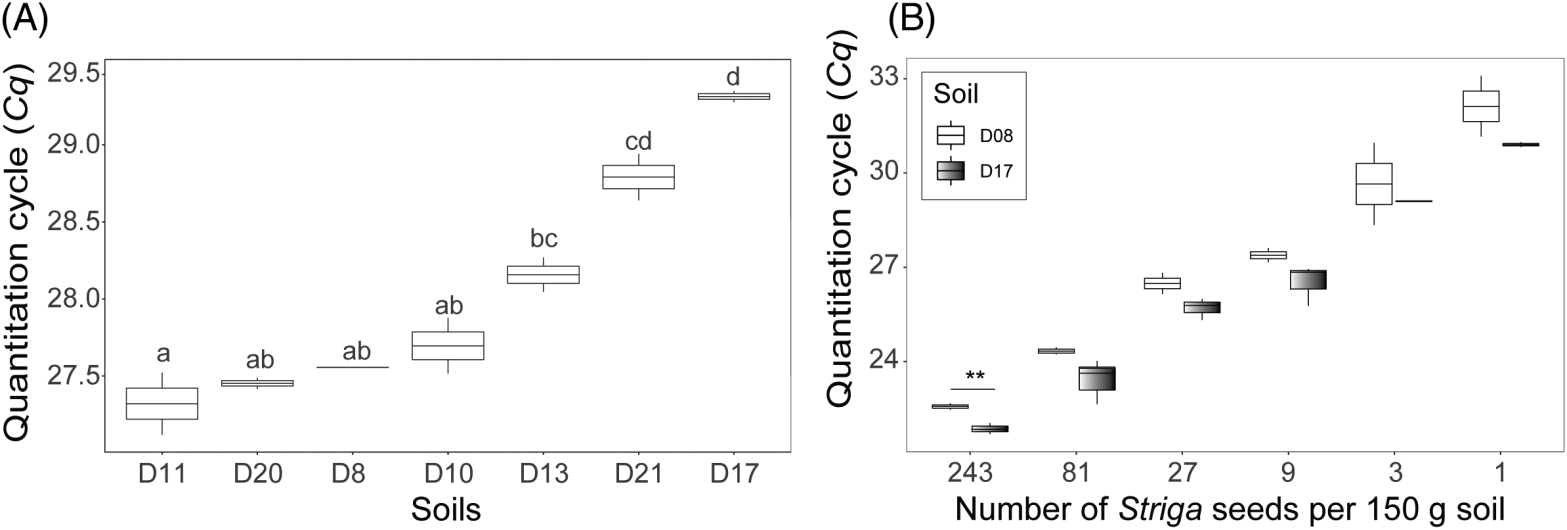
Influence of soil physicochemical properties on *Striga* seed detection. **(**A) qPCR detection of 65 *Striga hermonthica* seeds mixed into seven physicochemically different Dutch agricultural soils (D08, D10, D11, D13, D20, D21, D17). After mixing the seeds into these soils, total DNA was extracted and subjected to qPCR with primer set 14 (see Figure 1B). Different letters above the bars indicate a statistically significant difference (*p* < 0.05) between the *Cq* values of the seven soils; (B) qPCR detection of different *Striga* seed densities introduced into two physicochemically distinct Dutch agricultural soils (D08, D17). In contrast to the procedure used in panel a, soils amended with the *Striga* seeds were first treated with K_2_CO_3_ for size‐dependent separation of the *Striga* seeds from the soil matrix prior to DNA extraction. For both experiments, the mean *Cq* values (± SE) are shown for three biological replications and two technical replications per biological replication

The impact of soil physicochemical properties was assessed by introducing 65 *Striga* seeds into seven physicochemically different *Striga*‐free Dutch agricultural soils (Table S2). qPCR analysis on eDNA extracted from these ‘*Striga*‐spiked’ soil samples showed significant variation in the mean *Cq* value from 27.3 to 29.3 cycles (Figure 2A). However, no significant correlations were detected between the mean *Cq* value of the soils and the associated measured physicochemical attributes (*p* > 0.05).

To minimise interference of soil physicochemical properties, we then tested if separation of *Striga* seeds from the bulk soil prior to eDNA extraction and qPCR could improve the sensitivity of *Striga* detection and quantification. To this end, we adopted a density‐dependent K_2_CO_3_ separation of the *Striga* seeds from the soil matrix followed by successive sieving through two filters having mesh sizes of 425 and 75 μm, respectively. The *Striga* seeds, smaller soil particles and organic debris that were retained on the 75 μm filters were collected and dried at 35°C for 48 h and subjected to DNA extraction. This procedure reduced the soil volume by on average 99.7% for two physicochemically different Dutch soils tested and made it possible for the samples to be directly processed with the widely available DNA extraction kits.

Next, we introduced increasing densities of *Striga* seeds in soils D08 (sandy) and D17 (clay) at final densities of 0, 1, 3, 9, 27, 81 and 243 seeds per 150 g of soil and processed these soil samples as described above. Results of the qPCR analysis revealed that even a single *Striga* seed introduced into 150 g of soil sample can be detected by qPCR in both soil types (Figure 2B). Furthermore, for all the seed densities but density 243, the mean *Cq* values were not statistically different between the two soils (clay, sand) (Figure 2B).

### 3.3 Optimising quantification of *Striga* seeds in agricultural soil

**FIGURE 3:**
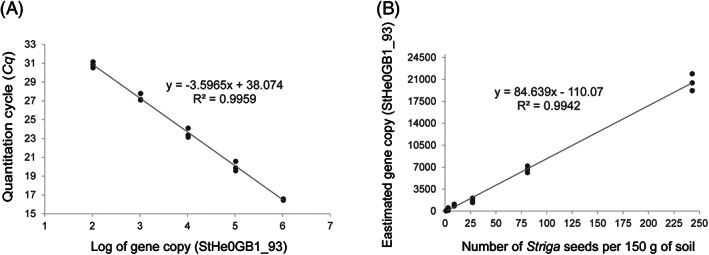
Standard curves to quantify *Striga* seeds in soil. (A) Relationship between the *Cq* values obtained in qPCR analysis of plasmid DNA containing the *Striga* marker gene StHe0GB1_93 (gene 5, Figure 1A) and the logarithm of the gene copy number. For each log gene copy number, three replicates were used in qPCR; (B) relationship between different *Striga hermonthica* seed densities mixed into agricultural soil and the estimated gene copy number. For each *Striga* seed density, three biological replications and two technical replications per biological replication were used. For each of the panels, a linear regression analysis was performed as shown in the equation including R^2^ values

For accurate quantification of the *Striga* seedbank in naturally infested field soils, two standard curves were generated. The first standard curve was generated from the 5‐point 10‐fold serial dilution (0.5 pg/μl to 0.00005 pg/μl) of the purified recombinant plasmid DNA (rpDNA) of the marker gene (StHe0GB1_93) cloned into the pGEM®‐T Easy vector. Mean *Cq* values of 16.51 and 30.82 were calculated for the highest (0.5 pg/μl) and lowest (0.00005 pg/μl) concentration, respectively, corresponding to 258 129 and 26 gene copies per μl^−1^ of single strand‐rpDNA according to Equation 1. The standard curve for StHe0GB1_93 is linear in the range tested (*R* ^2^ = 0.9959) with a slope of −3.5965 (Figure 3A). From the slope, an amplification efficiency of 89.69% was determined for StHe0GB1_93. Next, gDNA obtained from six *Striga* seed densities (1, 3, 9, 27, 81 and 243 seeds) introduced into 150 g of field soil D08 was subjected for qPCR and the mean *Cq* value corresponding to each seed densities was converted to gene copy based on the first standard curve. Then, to generate the second standard curve, the number of *Striga* seeds were plotted against the corresponding number of estimated gene copies (Figure 3B). The standard curve is linear in the range of *Striga* seed numbers tested (*R* ^2^ = 0.9942). Hence, this standard curve was then used for quantification of *Striga* seeds in naturally infested soil samples collected from sorghum growing fields in Ethiopia.

### 3.4 *Striga* seedbank density in naturally infested sorghum fields in Ethiopia

**FIGURE 4:**
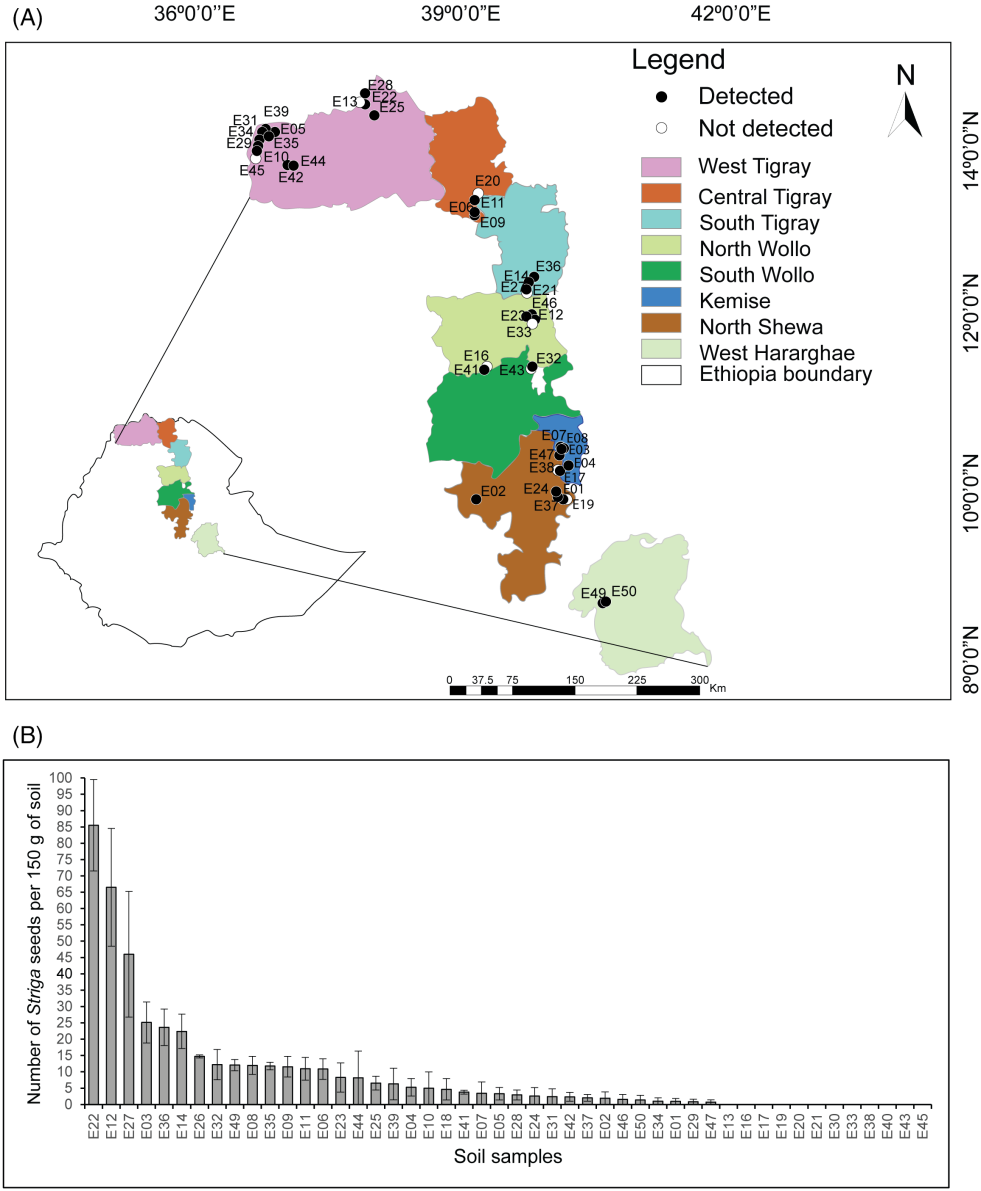
Geospatial mapping of *Striga* seedbank in sorghum belt of Ethiopia. (A) Map of Ethiopia showing the different sorghum growing agroecological zones and the agricultural field sites where the 48 soil samples (E numbers) were collected. If *Striga* seed is detected and quantified in 150 g of soil sample, the site is depicted with black dots otherwise with white dots. (B) Number of *Striga* seeds quantified by qPCR in 150 g of soil collected from each of these naturally infested field sites. Mean values (± SE) of three biological replicates (with two technical replicates per biological replicate) are shown

**FIGURE 5:**
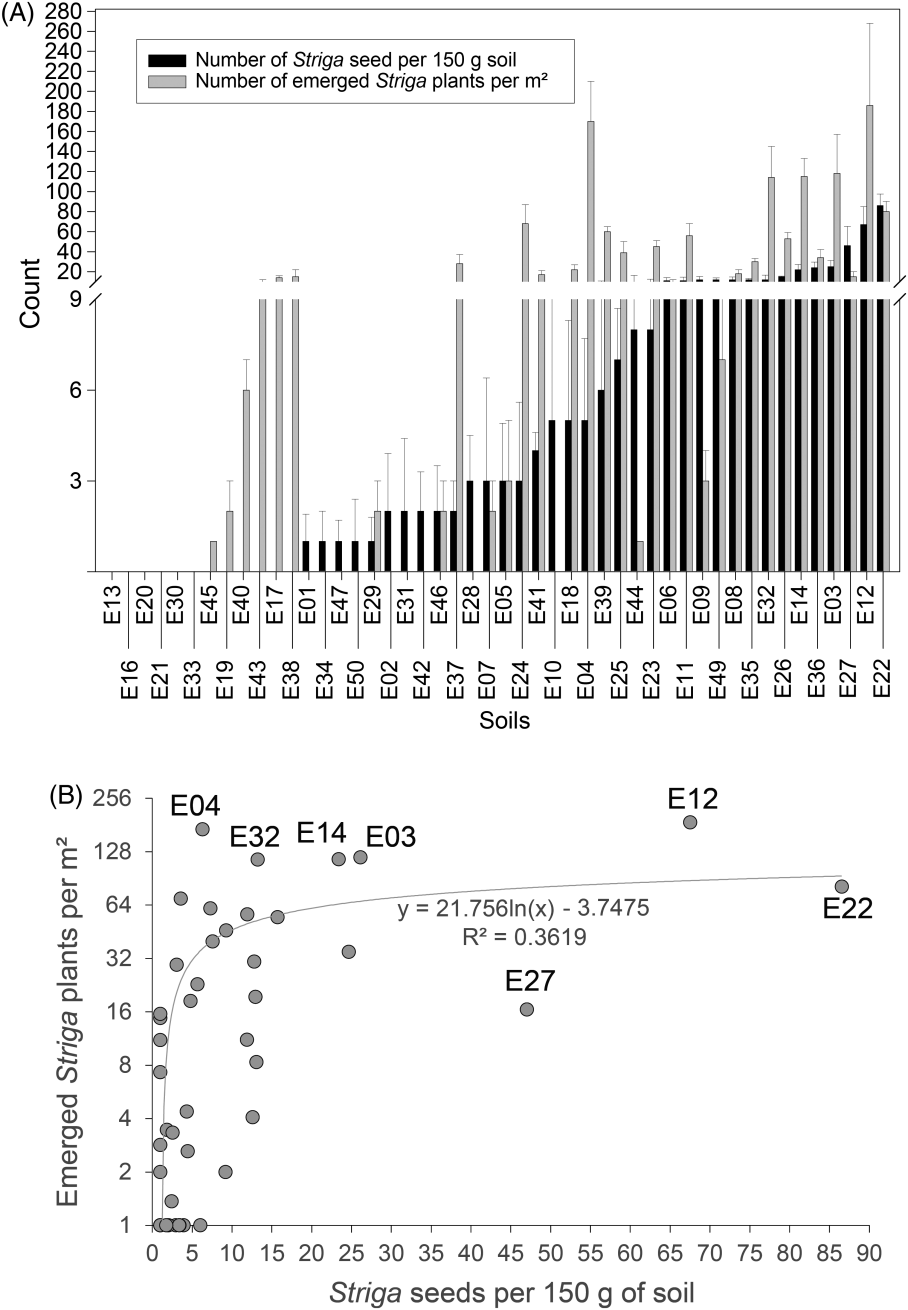
Relationship between *Striga* seedbank density and emerged *Striga* plants for 48 sorghum fields in Ethiopia (map shown in Figure 4A). (A) *Striga* emergence and *Striga* seed densities of 48 naturally infested sorghum fields in Ethiopia. (B) Non‐linear relationship between the number of emerged *Striga* plants per m^2^ and the number of *Striga* seeds detected per 150 g of soil sample. *Striga* emergence on the *Y*‐axis is shown on a log2 scale. For each sorghum field, the *Striga* seed density was quantified per 150 g of soil for three biological replicates as depicted in Figure 4B. *Striga* emergence was counted from four randomly chosen spots per field site and the number of sorghum plants counted per m^2^ was used to normalise the number of emerged *Striga* plants

Forty‐eight naturally infested soil samples (referred to as E01–E50, excluding soils E15 and E48) collected from sorghum fields from different agroecological zones in Ethiopia covering a trajectory along the sorghum belt of more than 1500 km were used to assess the variation in seedbank densities (Figure 4A). Using the method that was validated on artificially infested soils, we found substantial variation in *Striga* seed density among the 48 Ethiopian soil samples (Figure 4B and Table S1). The *Striga* seed densities were ranged from not detectable to 86 seeds per 150 g of soil sample, with soil samples E22, E12 and E27 harbouring the highest *Striga* seed densities with 86, 67 and 46 seeds per 150 g, respectively (Figure 4B). *Striga* seeds were not detected in soil samples of 12 Ethiopian sorghum fields (E13, E16, E17, E19, E20, E21, E30, E33, E38, E40, E43, and E45) (Figure 4B **)**. Furthermore, *Striga* seeds were detected in soil samples collected from push–pull fields (E01, E07, E49 and E50, respectively, having 1, 3, 12 and 1 *Striga* seeds per 150 g of soil) though no or low *Striga* incidence was observed in these fields during soil sampling (Figure 5A). On average, 75% of the soil samples were infested to a varying degree with *Striga* seeds. When looking into the geospatial distribution of the *Striga* seedbank in Ethiopian sorghum fields, most of the soils with relatively high *Striga* seed densities were collected from the Tigray region of Ethiopia. The majority of the samples that showed relatively low *Striga* seed densities were collected from sorghum growing areas of North Shewa. Whether the *Striga* seeds detected in ±75% the field soils tested in this study are still viable dormant or/and non‐dormant cannot be derived from the approach we developed here. Hence, future identification of marker genes that distinguish viable dormant from viable non‐dormant seeds will be needed to design primers that differentially amplify mRNA extracted from these seeds to obtain a more detailed insight into dormancy of the *Striga* seedbank.

### 3.5 Relationship between *Striga* seedbank and *Striga* incidence in sorghum fields

The relationship between *Striga* seed densities and field infestation was established to predict the risk for crop losses in different agroecological conditions and to test the efficacy of specific management practices. Our data did not fulfil the basic assumptions of the linear model and hence a logarithmic curve non‐linear regression was employed to assess the dependency of emerged *Striga* plants in the field on the number *Striga* seeds present in the soil (Figure 5B). Most of the data points fit to the trend line although some of the samples showed deviation from it.

## 4 DISCUSSION

Control of *Striga* remains challenging due to its high fecundity and seed survival rates in soil (Bebawi et al., 1984; Mourik, 2007). The estimated fecundity ranges from 3000 to 84 000 seeds per plant and seeds produced by 2–3 *Striga* plants per square meter are enough to replenish the seasonal seedbank losses caused by different biotic and abiotic factors (Andrews, 1945; Delft et al., 1997; Stewart, 1990; Webb & Smith, 1996). These extraordinary features contribute to the build‐up of the *Striga* seedbank in the soil after each successful completion of its life cycle. Currently, large‐scale quantification of the *Striga* seedbank in agricultural fields is a daunting task due to the lack of rapid and robust methods to detect and quantify these parasitic weed seeds in agricultural soils. Here, we present a methodology that allows accurate detection and quantification of the *Striga* seedbank in agricultural fields, which can be of paramount importance in the management of *Striga* in general and for understanding the seedbank dynamics in particular. By combining size‐based fractionation and successive sieving with qPCR‐based detection, our methodology minimises interference of physicochemical features of agricultural soils with eDNA‐based quantification techniques (Griffiths et al., 2000; Leite et al., 2014; Narayan et al., 2016; Schrader et al., 2012; Wilson, 1997). So far, density‐dependent separation of *Striga* seeds and lighter organic matter from heavy soil particles by K_2_CO_3_ and sucrose solutions followed by observation and counting of the seeds under stereomicroscope was used for the physical quantification of *Striga* seeds in field soils (Delft et al., 1997; Hartman & Tanimonure, 1991). Microscopic quantification of fine‐sized seeds of parasitic weeds requires less facilities and expertises as compared to marker‐based detection and quantification of the seedbank of parasitic weeds in agricultural soils, but is prone to a high degree of inaccuracy, is labour intensive and time consuming.

The majority of the earlier studies on small‐sized weed seed detection in soil primarily focused on the detection of different species but did not provide a methodology to quantify the seedbank (Kirilova et al., 2019; Portnoy et al., 1997; Rehms & Osterbauer, 2003; Román et al., 2007). Recent work on molecular detection and quantification of the parasitic weed *Orobanche cumana* seeds showed 0.001 ng/μl DNA as a limit of detection when gDNA was used for generation of the calibration curve (Aly et al., 2019). Our method of detection showed high specificity and ultra‐high sensitivity (up to 0.00005 pg/μl) to *Striga*‐associated DNA in a soil matrix. The high detection sensitivity obtained in our study with qPCR is most likely attributed to the use of plasmid DNA containing only the marker gene, which is devoid of other DNA and inhibitors from seed samples that can interfere with amplification of the target gene in qPCR. Moreover, the spiking study on different *Striga* seed densities in two different soils revealed that, when combined with qPCR, our methodology can detect even a single seed introduced in 150 g of soil. In the study by Delft et al. (1997), where *Striga* seeds were manually counted, the traditional flotation method followed by counting under microscope had a recovery of up to 85%. Hence, our approach substantially improved *Striga* seed recovery and provided a molecular confirmation of *Striga* seed presence. Furthermore, the analysis revealed that for all seed densities tested (1, 3, 9, 27, and 81 seeds, except density 243 seeds), the variation in *Cq* values was not significantly different between these two physicochemically different soils. The seed density range that showed consistent detection efficiency falls under the normal range of *Striga* seed density reported in soil samples of African countries (Delft et al., 1997; Hartman & Tanimonure, 1991; Smith & Webb, 1996; Visser & Wentzel, 1980).

The method was then adopted on soil samples collected from naturally infested sorghum fields and the analysis showed considerable variation in *Striga* seedbanks across the sorghum belt in Ethiopia. The *Striga* seed densities ranged from 0 to 86 per 150 g of soil sample. This seedbank density range is in line with previous studies that physically quantified *Striga* seed densities in agricultural field soils of different African countries. For instance, per 100 g of soil sample 0–75 seeds of *S. asiatica, S. hermonthica* and *S. gesnerioides* in Nigeria (Hartman & Tanimonure, 1991), 0–32 seeds of *S. asiatica* in South Africa (Visser & Wentzel, 1980) and 0–54 *S. hermonthica* seeds in northern Ghana (Sauerborn et al., 2003) were reported. However, our result is relatively lower than the *Striga* seed densities (23–297 seeds per 100 g of soil) that was recorded at two locations in western Kenya (Oswald & Ransom, 2001). There was substantial variation in the seedbank densities between the different regions of Ethiopia where the soil samples were collected. The majority of the fields with high seedbank density was located in the Tigray Regional State whereas those with low *Striga* seed densities were in the North Shewa zone. Here, we would like to emphasise that the terms ‘low’ and ‘high’ *Striga* seed densities are merely used to categorise the seedbank of our soil samples and this may not reflect the extent to which it poses an adverse effect on sorghum growth and yield. For example, if the seed density is presented per square meter of field soil assuming 300 kg topsoil per square meter, then the lowest seed density detected (1 seed per 150 g of soil sample) still corresponds to approximately 2000 *Striga* seeds per m^2^. Translating the seed densities detected per 150 g of soil to densities that are relevant at field scale suggests the persistence of a high *Striga* seedbank in multiple fields in the sorghum belt of Ethiopia and also may explain why soils with very low *Striga* seed densities still exhibit a relatively high *Striga* emergence per m^2^.

Determining the relationship between *Striga* seed densities and field infestation would be highly instrumental to predict the risk for crop losses in different agroecological conditions and to test the efficacy of specific management practices. However, establishing such relationships is difficult as these are highly dependent on the sorghum genotypes and management practices used by the farmers at the time of sampling and in subsequent cultivations (Rodenburg et al., 2006). Here, we performed a non‐linear regression analysis to assess the relationship between the number of emerged *Striga* plants in the sorghum fields and the *Striga* seed density in these field soils. We found a logarithmic relationship fitting the data of all 48 field soils best, confirming and extending earlier observations by Rodenburg et al. (2006). The asymptotic nature of this non‐linear relationship appears to make biologically more sense than a strict linear relationship considering intraspecific competition for infection sites, for outgrowth and/or emergence at increasing *Striga* seed densities. Looking into more detail in the non‐linear relationship, some of our soil samples showed significant deviation. For example, soil E04 showed high *Striga* incidence but low *Striga* seed density, whereas soil E27 showed high seed density but low *Striga* incidence (Figure 5B). The underlying mechanisms of these deviations are under investigation and can be due to soil physicochemical, host genotypic and/or soil microbiological attributes that act on the *Striga* seedbank or on *Striga* root infection. A previous study also showed that even in fallow fields, 1 year after the last harvest, a decrease of 62% in the number of seeds was recorded for the top soil fraction. This was not the case for samples originating from below a depth of 10 cm, possibly reflecting the decrease in microbial activity with soil depth (Delft et al., 1997). Soils E12 and E22 that showed high *Striga* seedbank density and high *Striga* incidence could be considered as soils conducive for *Striga*, whereas soil E27 can be considered as a potential *Striga* suppressive soil. Although this regression analysis might not provide a conclusive means to categorise field soils as *Striga* conducive or suppressive, it can serve as a lead to interrogate further these soils for *Striga* suppressive traits.

## 5 CONCLUSIONS

In conclusion, the molecular marker‐based methodology developed here is a first important step to screen large numbers of soil samples in SSA for early detection and quantification of *Striga* seeds and for generating an accurate infestation map. Furthermore, the methodology can be used to assess the impact of soil microbiological and physicochemical properties as well as different intervention strategies on *Striga* seedbank dynamics. The next challenge will be differentiating viable dormant and viable non‐dormant weed seeds to fine‐tune further the relationship between *Striga* seedbank dynamics and *Striga* incidence.

## CONFLICT OF INTEREST

We declare no conflicts of interest.

## Supporting information


TABLE S1
Table S2
Table S3

FIGURE S1
Figure S2

## Data Availability

The data that support the findings of this study are available in the supplementary material of this article. Furthermore, the raw data on the number of emerged Striga plants in sorghum fields are available for anyone who would like to consult them.
