# Supplementary material for: Molecular detection and quantification of the Striga seedbank in agricultural soils
Source: Weed Res. 2022 Apr 29;62(3):181–91. doi: 10.1111/wre.12535 (PMC9322021; doi:10.1111/wre.12535)
Supplement: Supplementary file 1 — TABLE S1 Table S2 Table S3 [file WRE-62-181-s001.docx]

**Table S1** List of primer sets used in developing molecular detection and quantification of *S.* *hermonthica* seeds in soil. For each primer set, the predicted PCR fragment length is given.

| **Marker gene** | **primer set** | **Primer sequence (5' 3')** | **PCR fragment length (bp)** |
| --- | --- | --- | --- |
| StHe0GB1_1 | 1 | CTGGTGGTCTCGTTGAGCTT | 145 |
|  |  | GAGTTAGAACCCGGCGAACA |  |
|  | 2 | GTTCGCCGGGTTCTAACTCT | 161 |
|  |  | CTAGCTCAGACGGACAACCG |  |
|  | 3 | CGTCTTCTCGAGGTGTGGTT | 170 |
|  |  | AAATTAGCGCCAAAGGCGAG |  |
| StHe0GB1_9 | 4 | ACTGCGATCTTGGCAGCAT | 115 |
|  |  | CAGTCCACTCACTCTTTGCC |  |
|  | 5 | TGGCAGCATCCATTGGTCAT | 157 |
|  |  | TGCATGTCGAGCAAAAGCAG |  |
| StHe0GB1_20 | 6 | GCAAAATGGGATCGTCTGGA | 111 |
|  |  | CAGCACGAAAGTTCTTCTGCC |  |
|  | 7 | CGAGGACAAAACTGAGGCCA | 200 |
|  |  | TTATACCGGGCCTCAAGGGA |  |
| StHe0GB1_76 | 8 | TCGGATAGCGACGTGGAAAG | 112 |
|  |  | ATTGTTGGGACCTCGGACTG |  |
|  | 9 | CGTTGGGCCCCGTATTACAT | 154 |
|  |  | AGACGTGCTAGCCGTAAACA |  |
|  | 10 | GAGGTCCCAACAATGCGAGA | 101 |
|  |  | ATGTAATACGGGGCCCAACG |  |
|  | 11 | GTGAGATTGACCTCGGGTGT | 100 |
|  |  | CCCAGCACTACCACTGAGTT |  |
| StHe0GB1_93 | 12 | AGACGGGCTGACTGGTAAAC | 276 |
|  |  | GCATTTTCTGGGGCTGATCG |  |
|  | 13 | TTGTTCGGCCCATCGGATTT | 70 |
|  |  | GTTTACCAGTCAGCCCGTCT |  |
|  | 14 | TCGGCCCATCGGATTTAGAA | 520 |
|  |  | TCGAGACGGGTTGAGTTAGG |  |

**Table S2** Chemical properties of Dutch agricultural soil samples used to assess the impact of soil physicochemical properties on DNA recovery and qPCR efficiency by introducing 65 *S. hermonthica* seeds in 100 mg of each soil.

| Soil sample | pH | Fe (mg/kg) | K (mg/kg) | Mg (mg/kg) | P (mg/kg) | S (mg/kg) | OM (%) | C (%) | N (%) |
| --- | --- | --- | --- | --- | --- | --- | --- | --- | --- |
| D08 | 6.87 | 0.076 | 181.542 | 109.681 | 16.226 | 1.141 | 2.808 | 1.936 | 0.155 |
| D10 | 7.49 | 0.024 | 97.074 | 70.883 | 1.117 | 2.956 | 2.924 | 2.622 | 0.157 |
| D11 | 7.28 | 0.112 | 68.772 | 56.435 | 5.432 | 1.174 | 3.485 | 1.491 | 0.159 |
| D13 | 7.55 | 0.044 | 207.126 | 122.521 | 3.401 | 7.568 | 7.033 | 3.816 | 0.338 |
| D17 | 7.75 | 0.015 | 87.171 | 74.542 | 1.029 | 2.272 | 4.232 | 2.631 | 0.148 |
| D20 | 7.30 | 0.101 | 234.447 | 299.632 | 2.688 | 6.975 | 8.003 | 2.877 | 0.363 |
| D21 | 5.56 | 0.821 | 58.819 | 484.438 | 4.856 | 24.700 | 20.589 | 9.295 | 1.042 |

**Table S3** Descriptive statistics of the number of *S.* *hermonthica* seeds detected per 150 g of soil in 48 samples collected from sorghum fields of Ethiopia.

| Soil sample | Striga seeds per 150 g of soil | Standard deviation | Standard error | Minimum | Maximum |
| --- | --- | --- | --- | --- | --- |
| E01 | 1 | 1.7 | 1.0 | 0 | 3 |
| E02 | 2 | 3.5 | 2.0 | 0 | 6 |
| E03 | 25 | 11.0 | 6.4 | 18 | 38 |
| E04 | 5 | 4.7 | 2.7 | 0 | 9 |
| E05 | 3 | 3.5 | 2.0 | 0 | 7 |
| E06 | 11 | 5.3 | 3.1 | 5 | 15 |
| E07 | 3 | 5.8 | 3.3 | 0 | 10 |
| E08 | 12 | 4.6 | 2.6 | 8 | 17 |
| E09 | 12 | 5.5 | 3.2 | 6 | 17 |
| E10 | 5 | 8.7 | 5.0 | 0 | 15 |
| E11 | 11 | 6.1 | 3.5 | 4 | 16 |
| E12 | 67 | 31.4 | 18.1 | 31 | 90 |
| E13 | 0 | 0.0 | 0.0 | 0 | 0 |
| E14 | 23 | 9.0 | 5.2 | 17 | 33 |
| E16 | 0 | 0.0 | 0.0 | 0 | 0 |
| E17 | 0 | 0.0 | 0.0 | 0 | 0 |
| E18 | 5 | 5.7 | 3.3 | 0 | 11 |
| E19 | 0 | 0.0 | 0.0 | 0 | 0 |
| E20 | 0 | 0.0 | 0.0 | 0 | 0 |
| E21 | 0 | 0.0 | 0.0 | 0 | 0 |
| E22 | 86 | 14.0 | 8.1 | 100 | 72 |
| E23 | 8 | 7.6 | 4.4 | 3 | 17 |
| E24 | 3 | 4.6 | 2.7 | 0 | 8 |
| E25 | 7 | 2.5 | 1.5 | 4 | 9 |
| E26 | 15 | 1.2 | 0.7 | 14 | 16 |
| E27 | 46 | 33.0 | 19.1 | 13 | 79 |
| E28 | 3 | 2.6 | 1.5 | 0 | 5 |
| E29 | 1 | 1.2 | 0.7 | 0 | 2 |
| E30 | 0 | 0.0 | 0.0 | 0 | 0 |
| E31 | 2 | 4.0 | 2.3 | 0 | 7 |
| E32 | 12 | 8.2 | 4.7 | 3 | 19 |
| E33 | 0 | 0.0 | 0.0 | 0 | 0 |
| E34 | 1 | 1.7 | 1.0 | 0 | 0 |
| E35 | 12 | 2.1 | 1.2 | 10 | 14 |
| E36 | 24 | 9.5 | 5.5 | 18 | 35 |
| E37 | 2 | 1.7 | 1.0 | 0 | 3 |
| E38 | 0 | 0.0 | 0.0 | 0 | 0 |
| E39 | 6 | 8.5 | 4.9 | 0 | 16 |
| E40 | 0 | 0.0 | 0.0 | 0 | 0 |
| E41 | 4 | 1.0 | 0.6 | 3 | 5 |
| E42 | 2 | 2.5 | 1.5 | 0 | 5 |
| E43 | 0 | 0.0 | 0.0 | 0 | 0 |
| E44 | 8 | 14.4 | 8.3 | 0 | 25 |
| E45 | 0 | 0.0 | 0.0 | 0 | 0 |
| E46 | 2 | 2.9 | 1.7 | 0 | 5 |
| E47 | 1 | 1.2 | 0.7 | 0 | 2 |
| E49 | 12 | 2.9 | 1.7 | 10 | 15 |
| E50 | 1 | 2.3 | 1.3 | 0 | 4 |
| Significance level | ≤0.0001 |  |  |  |  |
| CV (%) | 91.8 |  |  |  |  |
| LSD (0.05) | 13.7 |  |  |  |  |
