# Supplementary material for: Molecular detection and quantification of the Striga seedbank in agricultural soils
Source: Weed Res. 2022 Apr 29;62(3):181–91. doi: 10.1111/wre.12535 (PMC9322021; doi:10.1111/wre.12535)
Supplement: Supplementary file 2 — FIGURE S1 Figure S2 [file WRE-62-181-s002.docx]

**
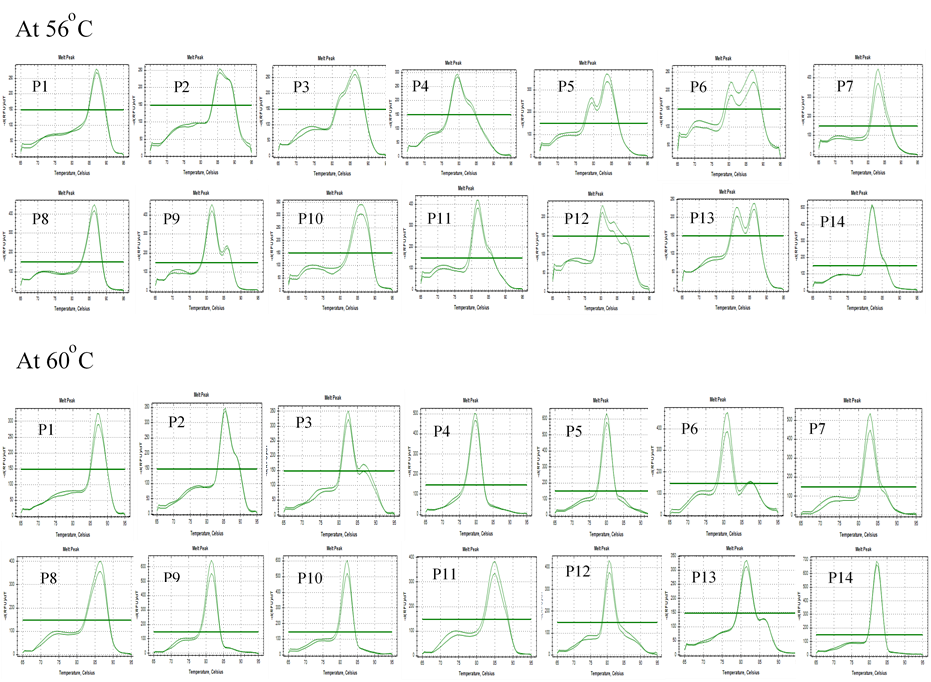
**

**Figure S1** Melting curves of the genomic DNA extracted from *S. hermonthica* seeds mixed into Dutch agricultural soil. qPCR analysis was performed with 14 different primer sets at two different annealing temperatures (see Figure 1 for details on the primer sets tested).


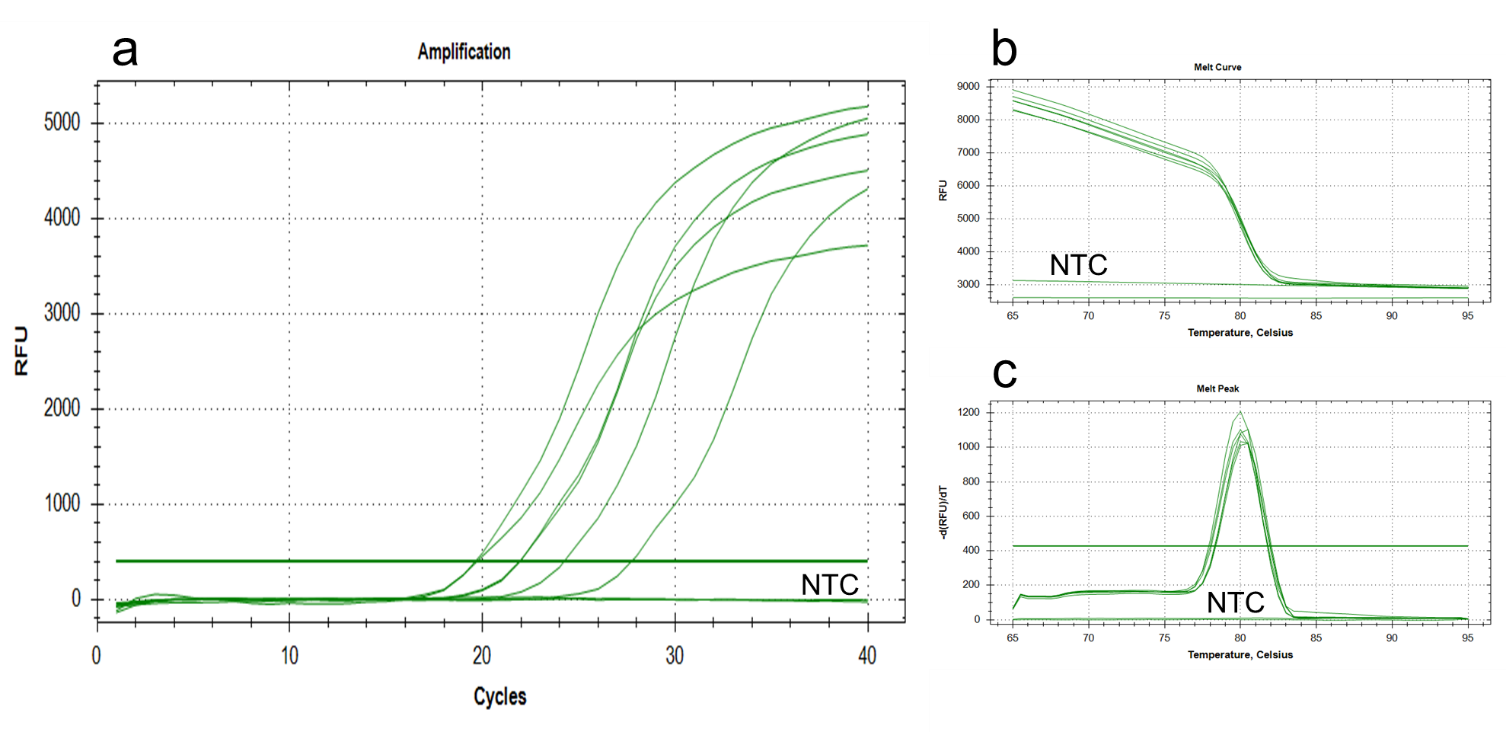


**Figure S2** qPCR detection of *Striga* seeds. (**a**) Detection of five *S. hermonthica* biotypes and one *S. asiatica* biotype collected from different regions of Ethiopia by qPCR. (**b**) Melt curve and (**c**) Melt peak associated with the detection of the samples by qPCR. The single peak corresponds to specific amplification of the target gene. NTC represents non-templet control.
